# Using Spectral Representation to Classify Proteins’ Conformational States

**DOI:** 10.3390/ijms19072089

**Published:** 2018-07-18

**Authors:** Seyed Majid Saberi Fathi, Jack A. Tuszynski

**Affiliations:** 1 Department of Physics, Faculty of Science, Ferdowsi University of Mashhad, Mashhad 9177948974, Iran; 2 Department of Oncology, University of Alberta, Edmonton, AB T6G 1Z2, Canada; jackt@ualberta.ca; 3 Department of Physics, University of Alberta, Edmonton, AB T6G 2E1, Canada; 4 Department of Mechanical and Aerospace Engineering, Politecnico di Torino, 10129 Torino, Italy

**Keywords:** protein, conformational states, spectral representation

## Abstract

Numerous proteins are molecular targets for drug action and hence are important in drug discovery. Structure-based computational drug discovery relies on detailed information regarding protein conformations for subsequent drug screening in silico. There are two key issues in analyzing protein conformations in virtual screening. The first considers the protein’s conformational change in response to physical and chemical conditions. The second is the protein’s atomic resolution reconstruction from X-ray crystallography or nuclear magnetic resonance (NMR) data. In this latter problem, information is needed regarding the sample’s position relative to the source of X-rays. Here, we introduce a new measure for classifying protein conformational states using spectral representation and Wigner’s D-functions. Predictions based on the new measure are in good agreement with conformational states of proteins. These results could also be applied to improve conformational alignment of the snapshots given by protein crystallography.

## 1. Introduction

Proteins are flexible biomolecules whose conformations change in response to physical and chemical conditions. However, protein functions depend on their specific conformation. A protein’s 3D conformation is determined by the primary sequence of amino acids [1]. Knowing the relation between protein fluctuational motion and its sequence can be used to design de novo dynamics of proteins and provide putative conformations for drug targets by accessing information about transition states [1]. Predicting protein motion is a computational challenge. Some conformational search algorithms use a coarse graining representation of the protein molecule [2,3,4] and employ search methods such as elastic network modeling [5,6,7,8,9,10], morphing [11,12], or Normal Mode Analysis [13,14]. Other methods apply motion planning algorithms used in robotics to find a pathway to protein conformations [15,16,17]. Other methods combine coarse graining and motion planning [18]. Finding a quantitative measure that can be reliably used to classify conformational states of a protein is important for both virtual screening in general and structure-based drug design (SBDD) in particular [19]. The most frequently used experimental method to determine protein conformation is X-ray crystallography [20], which is only applicable when the given protein can be crystallized. The pattern of diffraction of an X-ray is used to determine the protein’s atomistic structure. One of the important factors in protein-protein and protein-ligand binding is geometry matching. 

In this paper, we introduce a simple mathematical measure to classify protein conformations, which is defined in reciprocal space and uses the corresponding structure factors of the given protein. Reciprocal space has advantages over real space since we can compare two proteins with different numbers of atoms using the same dimensions in reciprocal space, i.e., reciprocal space is independent of protein sizes [21]. This can allow for the classification of different conformations from the data obtained, e.g., by X-ray free electron laser (XFEL), of a protein with unknown structure before reconstructing it. One of the issues with XFEL is known as the diffract-and-destroy problem [22,23,24] in which X-rays irradiate during a few femtoseconds a sample before it is destroyed, so that different samples are needed to have a sufficient number of snapshots to reconstruct a protein as a single nanoparticle. On the other hand, each protein sample could have different orientations, conformations, or configurations in other samples. Thus, information about the conformation of snapshots is necessary for a proper reconstruction of the protein data from its images [25].

## 2. Results and Discussion

To prove the reliability of our method based on MED we examine below three different synthetic datasets. The first dataset is a simple shape-simulated dataset of non-structured 12-mer peptides, the second is a simulated protein dataset and the third dataset is a simulated X-ray diffraction dataset of ADK.

**Figure 1:**
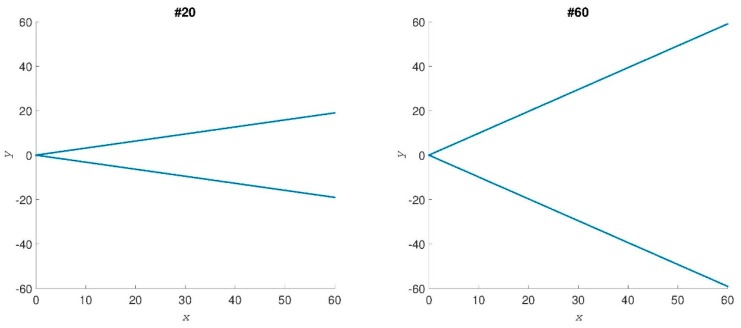
Two samples of test conformational shapes. The axes are *x* and *y* positions of the points.

**Figure 2:**
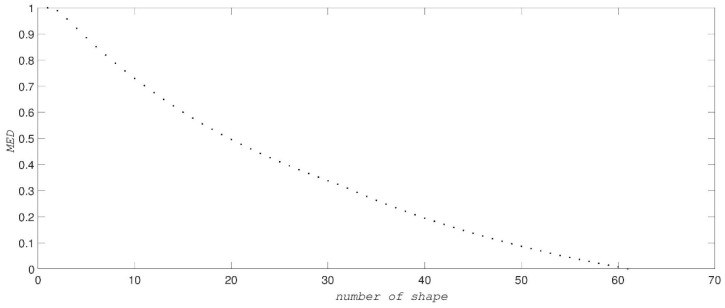
The MED values for the simple shape dataset. The abscissa gives the number of test shapes.

*Simple shape dataset*. To show the robustness and reliability of MED we first use a simulated conformational dataset for shapes ranging from an open to a closed conformation, and two examples out of 61 cases analyzed are shown in Figure 1. Formula (1) gives the set of $\left\{C_{lmn}\right\}$ for each of these conformations. We can compute MED by selecting the largest eigenvalue of the “d” matrix from Formula (3) for each shape. The MED values are shown in Figure 2, where the abscissa gives the number of shapes. Shape #1 is totally closed and shape #61 is the most opened shape. MED values decrease when the conformations of shapes vary from close to open. 

*Protein dataset*. The MED values obtained confirm the prediction regarding the corresponding conformations. We now perform the second test by examining conformations between quasi-real data to the protein structures, and use the data generated by MOE software of Chemical Computing Group Inc. (available online: http://www.chemcomp.com/). We have created a library of 4-mer peptides for surfaces with different characterization, which is the starting point for finding longer (more closed) peptides, which can recognize and bind to a given surface with high affinity and specificity. These data have been obtained by molecular dynamics (MD) simulations for 4-mer peptide sequences. We have first computed structure factors and then produced the distance matrix in order to find the MED for all 44 conformations of non-structured 12-mer peptides (called s1, s14, s16 and s31). For example, we see that the MED for s14-3 is $\mu_{i}=0.6827$ and it is closed, while for s1-4 peptide MED is $\mu_{j}=0.3155$ and it is open. In Table 1, we present the MED values for all 44 conformations of non-structured 12-mer peptides (which are called s1, s14, s16, and s31).

**Figure 3:**
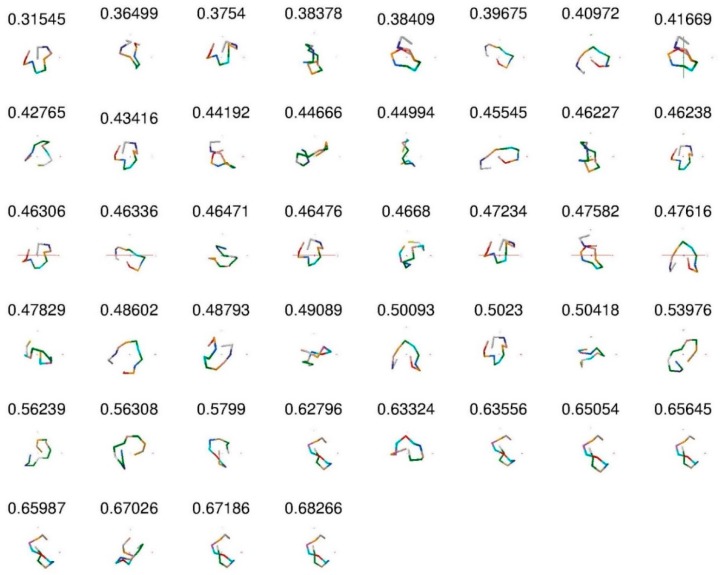
The 44 peptides and their MED values. We see that peptides are more closed when MED increased.

Figure 3 shows the 44-peptide backbone images with their MED values sorted from small to large values. The larger MED corresponds to the more closed (longer) peptides. This figure is consistent with closed and open conformation assignments. 

*Diffraction snapshots dataset*. One of the main issues in the reconstruction of the biological systems from X-ray scattering snapshots is that some information, which is known in crystallography, is unknown to the biological system, e.g., the structure’s orientation. Conversely, information exists in the biological system’s reconstruction, e.g., conformation, which does not exist in crystallography, since only one sample may be used in crystallographic measurements while a biological system such a protein may involve many samples in a statistical ensemble. Hence, a large number of XFEL snapshots are combined with different conformations and unknown orientations. Thus, before reconstruction, we should cluster these data and then use the reconstruction methods that use X-ray diffraction patterns such as crystallography. 

**Figure 4:**
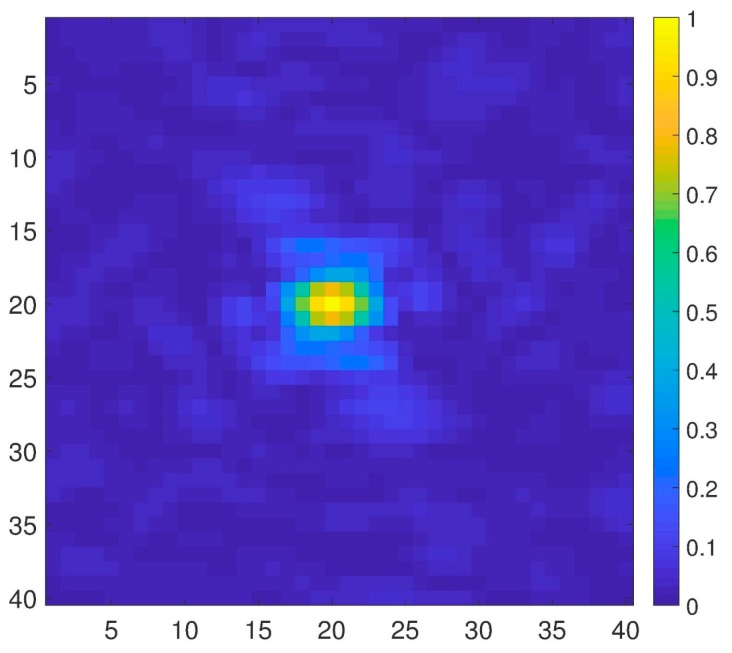
An X-ray diffraction pattern of ADK [26].

**Figure 5:**
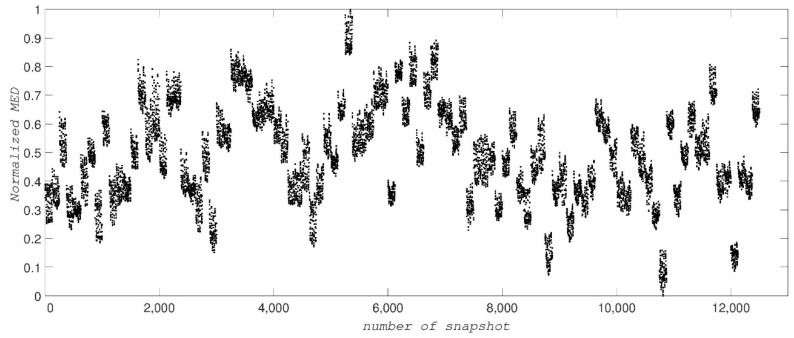
The normalized MED values for 12,500 snapshots of ADK showing 100 islands for the 100 different conformational states.

In the third test of our method, we use the dataset simulated by MD of the unfolding of ADK [26], which contains 12,500 diffraction snapshots simulated from 100 conformations, with each conformation assuming 125 orientations about one axis. The coordinates of ADK [27] from *E. coli* in the open state (Protein Data Bank entry: 4AKE) were placed in a spherical droplet of water and simulated using NAMD software [27]. Figure 4 shows a typical diffraction snapshot of this dataset. We should first complexify this dataset by taking successively the inverse Fourier and then its Fourier transform for each snapshot. Having the complex form of a snapshot, we then obtain a distance matrix and its largest eigenvalue, MED. Figure 5 illustrates the MED for these 12,500 snapshots. These values are separated among a hundred islands (conformations) so that each island with 125 snapshots represents a conformation. 

To perform the required computations, one needs several hundred megabytes of memory and a computation time of about 950 s for all of 12,500 snapshots of this dataset by using a CPU-i3 with 4GB RAM. The three tests show that MED is a reliable and low-computational cost measure for the prediction of conformational states of proteins. It is especially useful to cluster these patterns before having the protein’s structure obtained from X-ray diffraction in the dataset. 

In the time-resolved X-ray crystallography methods, for structure reconstruction we need to have the crystal’s orientation in crystallographic measurements. In the corresponding biological assays, due to significant effects of radiation damage one needs to use replicate samples [28]. Hence, we need more information on the protein system, such as its conformational states. Hence, we must classify snapshots in conformational states, orientations, etc., in order to solve the problem. There exist some methods to modify diffraction experiments such as probe-pump-probe, etc. [29,30,31,32]. MED can classify conformational states using snapshots obtained from X-ray diffraction from biological samples and it does not require the numbers of atoms to be the same in all cases.

Working in reciprocal space with structure factors has substantial advantages such as dependence on the topology and geometry of proteins and independence of the protein’s size [21]. Using the spectral representation for classifying protein shapes has led to the introduction of a new measure, MED. Here, MED has been used on three different sets. MED-based prediction has been shown in good agreement with simulated data. MED can be used to study protein dynamics as a spectral representation of time evaluation, and protein conformational state classification in the drug discovery, reconstruction of protein structure from X-ray data, and also biological applications. 

## 3. Method

The weighted similarity value (WSV) is an alternative to root-mean-squared deviation (RMSD) that allows a similarity comparison between two proteins [21,33]. WSV has been defined as an illustration of the Wigner-D function [34] by expanding the protein’s shape functions. Any compact supported function can be expanded in terms of Wigner-D functions, $D_{lmn}\left(\alpha ,\beta ,\gamma \right)$, such as the protein’s shape function, f(α,β,γ), with α,β, and γ as the Euler angles. Here, f(α,β,γ) is defined as the molar mass of an atom located on the (α,β,γ) coordinate set and if there is no atom there, it is assumed to be zero [33]. The expansion coefficients, $C_{lmn}$s, are unique for a given function, f(α,β,γ), and are obtained by:

(1) $$C_{lmn}=\frac{\left(2l+1\right)}{8\pi^{2}}\int \int \int f\left(\alpha ,\beta ,\gamma \right)\;{D_{lmn}}^{*}\left(\alpha ,\beta ,\gamma \right)\;\sin\beta \;d\beta \;d\alpha \;d\gamma$$

It has been shown [35,36] that $C_{lmn}$ correspond to elements of the 3D Fourier transform of (α,β,γ). In crystallography, the coefficient of the Fourier transform of a crystal shape function is called a structure factor [37], hence $C_{lmn}$ represent the protein structure factors. Note that two shapes of different sizes in real space have the same dimensions in reciprocal space in spite of having different numbers of atoms [21]. 

In this paper, we use another advantage of working in reciprocal space, i.e., replacing a time series by spectral representation in reciprocal space as a set of eigenvectors. This is a result of the Fourier transform of the time derivative of a function, which is equal to the spectral of the Fourier transform of the function:

(2) $$\;\hat{\left(\frac{\partial g}{\partial t}\right)}=i\;\omega \;\hat{g}$$

where the hat ⋀-symbol over a function indicates a Fourier transform. The set: $\left\{\omega \right\}=\left\{\omega_{1},\omega_{2},\cdots \right\}$ represents a spectral representation corresponding to the time series set $\left\{t\right\}=\left\{t_{1},t_{2},\cdots \right\}$. In fact, one set of temporally connected conformations would give a single set of eigenvectors, i.e., one spectral representation. Therefore, to observe change in conformation, such as during an unfolding of a protein, one can divide the total event into shorter time-spans, each representing a time-average conformation. 

$C_{lmn}$s belong to the complex space and can be embedded in the $\left(N_{R}\times 2\right)$-dimensional Euclidean space as the following: $\left\{\mathrm{Real}\left(C_{lmn}\right),\;\mathrm{Imaginary}\left(C_{lmn}\right)\right\}$ = $\left\{C_{i1},\;C_{i2}\right\},\;\mathrm{and}\;\left(i=1,2,\cdots N_{R}\right)$, where $N_{R}$ is the number of $C_{lmn}$s defined by choosing $L_{max}$, i.e., maximum l in $D_{lmn}\left(\alpha ,\beta ,\gamma \right)$. Note that each conformation has one corresponding structure factor. Then, we define distance matrices, {d}, as the distance matrix between elements of structure factor related to a conformational state. By obtaining the set eigenvalues of d matrix, i.e., {a}, we introduce a new measure to compare the different conformations of a given protein by computing the largest eigenvalue:

(3) $$\;\mu_{i}=\max\left\{a\right\}$$

Here $\mu_{i}$ is the maximum eigenvalues of the distance matrix related to the *i*th protein’s conformational state. If $\mu_{i}$ is larger than $\mu_{j}$, it signifies a more closed conformation of the protein, since length a in real space is proportional to 1/a in reciprocal space, where μ as a vector (column matrix) with an N-element for N-conformational states and we call it the “maximum eigenvalue of distance matrix” (MED). The MED value depends on the protein’s structure and it is unbounded. Thus, if two protein structures are not temporally connected, the differences between the corresponding MED-values are not significant. To compute MED for X-ray scattering from a single nanoparticle we find the distance matrix by complexifying snapshots, which implies taking its inverse Fourier and then Fourier transforming it.

The maximum eigenvalue plays an important role in data analysis [38,39,40,41], because the eigenvectors define a basis for a vector and the corresponding eigenvalues are vector projection magnitudes in this basis set. Thus, it implies the most effective base (eigenvector). In the dimensional reduction process the largest eigenvalue indicates the most effective change occurring in its corresponding eigenvector. This change could represent conformation, rotation, translation, dilation, etc. [26,42,43,44,45]. For protein shapes, conformation is the most effective parameter in the snapshots, which is obtained by X-ray diffraction, because a diffraction pattern depends on the distance between two atoms, thus, the largest eigenvalue should best describe this change. Here, we use the reciprocal distance matrix between two different conformations and find the largest eigenvalues, which describe the important conformational change between the two shapes. 

## Figures and Tables

**Table 1:** The names of the different conformations of peptides generated by MOE software and the corresponding values of their MED. Larger numbers correspond to a more closed (longer) peptide conformation.

Peptide Name	MED
s1-17	0.41669
s1-18	0.44192
s1-21	0.3754
s1-1	0.46476
s1-10	0.46306
s1-11	0.46238
s1-12	0.5023
s1-13	0.39675
s1-14	0.47616
s1-15	0.48602
s1-16	0.43416
s1-19	0.45545
s1-2	0.40972
s1-20	0.48793
s1-3	0.50093
s1-4	0.31545
s1-5	0.47582
s1-6	0.46336
s1-7	0.38409
s1-8	0.36499
s1-9	0.47234
s14-1	0.65987
s14-10	0.63324
s14-11	0.62796
s14-2	0.49089
s14-3	0.68266
s14-4	0.67186
s14-5	0.63556
s14-6	0.67026
s14-7	0.5799
s14-8	0.65054
s14-9	0.65645
s16-1	0.46471
s16-2	0.56308
s16-3	0.46227
s16-4	0.53976
s16-5	0.44666
s16-6	0.38378
s16-7	0.56239
s31-1	0.47829
s31-2	0.50418
s31-3	0.4668
s31-4	0.44994
s31-5	0.42765

## Abbreviations

MED	Maximum Eigenvalue Of Distance Matrix
